# Draft Genome Sequence of *Porphyromonas gingivalis* Strain SJD2, Isolated from the Periodontal Pocket of a Patient with Periodontitis in China

**DOI:** 10.1128/genomeA.01091-13

**Published:** 2014-01-02

**Authors:** Dali Liu, Yanbin Zhou, Mariko Naito, Hiromichi Yumoto, Qiang Li, Yoichiro Miyake, Jingping Liang, Rong Shu

**Affiliations:** Department of Periodontology, Ninth People’s Hospital, Shanghai Jiao Tong University School of Medicine, Shanghai Key Laboratory of Stomatology, Shanghai, Chinaa; Division of Microbiology and Oral Infection, Department of Molecular Microbiology and Immunology, Nagasaki University Graduate School of Biomedical Sciences, Nagasaki, Japanb; Department of Conservative Dentistry, Institute of Health Biosciences, The Tokushima University Graduate School of Dentistry, Tokushima, Japanc; Shanghai Majorbio Bio-pharm Technology Company Limited, Shanghai, Chinad; Department of Oral Microbiology, Institute of Health Biosciences, The Tokushima University Graduate School of Dentistry, Tokushima, Japane; Department of Endodontics, Ninth People’s Hospital, Shanghai Jiao Tong University School of Medicine, Shanghai Key Laboratory of Stomatology, Shanghai, Chinaf

## Abstract

*Porphyromonas gingivalis* strain SJD2 was isolated from subgingival plaque of a patient in China with chronic periodontitis. Here, we report the draft genome of this strain, with a size of 2,328,850 bp, average G+C content of 48.3%, and 2,020 predicted protein-coding sequences.

## GENOME ANNOUNCEMENT

*Pophyromonas gingivalis* is a black-pigmented bacterium from the phylum *Bacteroidetes* and is a major pathogen in periodontal disease (1). The complete genomic sequences of *P. gingivalis* strains W83, ATCC 33277, and TDC60 have been available (2–4). *P. gingivalis* SJD2 was obtained from subgingival dental plaque of a patient in China with chronic periodontitis (5). The 16S rRNA sequence of this strain showed 99% identity with those of type strains of W83 and ATCC 33277, as well as with clinical strain TDC60 (2). In addition, this strain was shown to have high virulent properties comparable with those of the type strain of W83 in a mouse abscess model.

The whole-genome sequence of *P. gingivalis* SJD2 was generated with short-insert DNA (496 bp) libraries using the high-throughput sequencing Illumina HiSeq 2000 platform (BGI, Shenzhen, China), which generated 100-bp paired-end sequences at a coverage of 100×. The resulting 257.5-Mb raw data were cleaned by removing adapter, low-quality reads, poly-N, and error pair-end reads. Quality-trimmed reads (Sanger Q, ≥20, 91.8% of total reads) were then assembled with SOAP*denovo* version 1.04 (http://soap.genomics.org.cn/soapdenovo.html). The draft genome, as submitted to GenBank, is 2,328,850 bp in length and comprises 140 contigs (>300 bp in size), with a mean contig size of 16,635 bp, a median size of 32,167 bp, and a maximum length of 85,472 bp. The mean G+C content of the genome is 48.3%.

Gene prediction was performed using the Prokaryotic Genome Automatic Annotation Pipeline (PGAAP) provided by National Center for Biotechnology Information (NCBI). A total of 2,021 protein-coding sequences (CDSs) and 51 structural RNAs, together with 64 pseudogenes and 3 clustered regularly interspaced short palindromic repeats (CRISPRs), were predicted. Each CDS was then reviewed manually for the presence of a start codon and a potential ribosome-binding Shine-Dalgarno (SD) sequence that should be related to a part of an AGAAAGGAGG(T) sequence, or an AANGA sequence with a sufficient size of 6 to 3 nucleotides (3). Three hundred four CDSs without SD sequences were performed with the homology search through the public protein database from NCBI using the BLASTp program (http://blast.ncbi.nlm.nih.gov/Blast.cgi?CMD=Web&PAGE_TYPE=BlastHome). One CDS that has a size of >180 bp showing an e-value of homology >e-10 was excluded from the 2,021 CDSs. Finally, 2,020 CDSs were predicted, and the details were given in the GenBank database.

Overall, BLASTp analysis with the complete genome sequences of W83, ATCC 33277, and TDC60 under 70% of identity covered 50 to 150% length revealed that 13.7% CDSs (278 of 2,020 CDSs) in SJD2 are represented as strain specific, which is higher than the percentages (6.5 to 9.6%) of strain-specific CDSs in other strain genomes. The higher number of SJD2-specific CDSs suggests that strains isolated from a periodontal pocket of Chinese patients with chronic periodontitis may have distinct genes. Further investigation of the genomes of this highly virulent clinical strain may yield more insights into the considerable pathogenicity of *P. gingivalis*.

### Nucleotide sequence accession numbers.

This whole-genome shotgun project has been deposited at DDBJ/EMBL/GenBank under the accession no. ASYL00000000. The version described in this paper is version ASYL01000000.
